# Bone quality assessment around dental implants in cone-beam CT images: effect of rotation mode and metal artefact reduction tool

**DOI:** 10.1093/dmfr/twaf003

**Published:** 2025-02-13

**Authors:** Lauren Bohner, Hian Parize, João Victor Cunha Cordeiro, Natalia Koerich Laureano, Johannes Kleinheinz, Ricardo Armini Caldas, Dorothea Dagassan-Berndt

**Affiliations:** Department of Cranio-Maxillofacial Surgery, University Hospital Muenster, Muenster, 48149, Germany; Department of Dentistry, Federal University of Santa Catarina, Florianópolis, SC 88040-370, 88040370, Brazil; Department of Cranio-Maxillofacial Surgery, University Hospital Muenster, Muenster, 48149, Germany; Department of Prosthodontics, School of Dentistry, University of São Paulo, São Paulo, 05508000, Brazil; Department of Dentistry, Federal University of Santa Catarina, Florianópolis, SC 88040-370, 88040370, Brazil; Department of Oral Pathology, Federal University of Rio Grande do Sul, Porto Alegre, 90650970, Brazil; Department of Cranio-Maxillofacial Surgery, University Hospital Muenster, Muenster, 48149, Germany; Department of Dentistry, Federal University of Santa Catarina, Florianópolis, SC 88040-370, 88040370, Brazil; Center for Dental Imaging, University Center for Dental Medicine Basel UZB, University of Basel, Basel, 4058, Switzerland

**Keywords:** cone-beam CT, dental implants, artefacts

## Abstract

**Objectives:**

The purpose of this study was to evaluate how artefacts caused by titanium and zirconia dental implants affect the bone quality assessment in CBCT images. The effect of scan mode and the use of metal artefact reduction (MAR) algorithm on artefacts suppression were taken in consideration.

**Methods:**

Titanium and zirconia dental implants were installed in porcine bone samples and scanned with two CBCT devices with adjustments on scan mode and with the use of MAR. The control group consisted of bone sample without implant and scanned with full-rotation scan mode without MAR. Artefacts extension and bone quality around implants were measured by deviation of grey values and bone histomorphometry measurements (trabecular volume fraction, bone specific surface, trabecular thickness, and trabecular separation), respectively. Mean difference among groups was assessed by within ANOVA with Bonferroni correction. Correlation between bone quality measurements acquired in the experimental and control groups was assessed by Spearman correlation test (*α* = .05).

**Results:**

No statistical difference was found for artefacts extension in images acquired by half and full-rotation modes (*P* = .82). The application of MAR reduced artefacts caused by titanium and zirconia dental implants, showing no statistically significant difference from the control group (titanium: *P* = .20; zirconia: *P* = .31). However, there was no correlation between bone quality measurements (*P* < .05).

**Conclusions:**

Bone quality assessment was affected by the presence of artefacts caused by dental implants. Rotation mode did not affect the appearance of artefacts and bone qualitative measurements. MAR was able to decrease artefacts, however, it did not improve the accuracy of bone quality measurements.

## Introduction

Cone-beam CT (CBCT) is widely used for the 3D jawbone assessment due to its high resolution and image quality. CBCT has been adopted not only for diagnosis, but also for dental implant planning or monitoring of peri-implant health. In many cases, the jaw bone assessment has to be evaluated surrounding dental implants.^1–3^

However, the assessment of peri-implant bone in CBCT images might present some limitations. It is well known that dental materials with high atomic number, as dental implants, generate unwanted effects on CBCT images, as metal streak, beam hardening, or scattering artefacts, which may hamper peri-implant bone assessment and diagnosis.^3^^,^^4^ Therefore, studies attempted to determine scanning protocols taking in consideration the ALADA (as low as diagnostically acceptable) principle. In this regard, adjustments of CBCT parameters, as rotation mode, and application of artefact reduction algorithms, have been proposed to generate an image with diagnostic quality and reduced radiation exposure.^5^^,^^6^

CBCT devices offer a scanning protocol mode with half rotation arc of 180°, followed by a 360° image reconstruction through algorithms. This scan mode is related to a low number of basis images, resulting in a subsequent reduction in radiation dose in detriment of image quality. Nonetheless, it represents a reliable alternative for the assessment of peri-implant bone.^6–11^

A second approach to reduce artefacts is the metal artefact reduction (MAR) algorithm, which suppress artefacts by reducing the variability of grey values (GV). For instance, some MAR algorithms take in consideration a threshold value. Any GV denser than this threshold will be correct to reduce artefacts, increasing diagnostic accuracy.^12–14^ However, it also influences the morphometric assessment of bone tissue.^15^ Controversial results have been reported in literature regarding the use of MAR for peri-implant bone assessment.^16–19^ Differences in MAR algorithms and CBCT scanner could be the source of such inconsistency.^20^

Although the appearance of dental implants artefacts is well documented in literature, fewer studies reported the influence of artefacts on peri-implant bone assessment.^21^ Furthermore, zirconia implants have been increasingly used in clinical practice, however they lead to more artefacts than titanium implant due to the high atomic number, further requiring imaging protocol adjustments which can influence diagnostic accuracy.^20^

Therefore, the purpose of this study was to investigate how artefacts caused by titanium and zirconia dental implants affect the bone assessment in CBCT images. In addition, the effect of rotation mode and the use of MAR on artefacts suppression were evaluated.

## Methods

Sample size was calculated based on a pilot study (*n* = 3) with the software G*Power (University Düsseldorf). Based on a *F*-test, and considering the artefacts assessment as the primary outcome, six samples (*n* = 6) per group were required to achieve an effect size of 0.57 µm with a power of 80% and a significance threshold set of 0.05.

### Specimen preparation

Porcine ribs were commercially acquired in a local butchery. Bone specimens measuring 4 cm in extension were prepared with a milling cutter after removal of soft tissue. Following manufacturer instructions, for each sample a dental implant bed was prepared and dental implants were installed. Six titanium (Bone Level, Institute Straumann AG, Basel, Switzerland) and six zirconia (Pure Ceramic, Institute Straumann AG, Basel, Switzerland) dental implants were used in this study (Appendix S1). Soft tissue was simulated by inserting the samples individually on plastic sample collection tubes filled with ultrasound gel (SHR Germany GmbH, Hilden, Germany).

### Image acquisition

After bed preparation and prior to dental implants placement, bone samples were individually scanned using a micro-computed tomography (µCT) device (SkyScan 1272; Bruker Physik GmbH, Billerica, United States) for methodological purposes. The following parameters were used: 80 kV, 125 mA, 16 µm of voxel size. The µCT images were adopted as a digital reference data set of peri-implant bone, which was used to guide the trabecular bone segmentation.

For CBCT scans, two devices (3D Accuitomo 170, Morita, Saitama, Japan; ProMax 3D max, PlanMeca, Helsinki, Finland) were configured with standard settings (90 kV, 8 mA, and 0.150-0.160 mm voxel size). Each sample was scanned twice, before and after dental implant placement. The CBCT scans of samples without dental implants were used as a control group.

**Figure 1.**
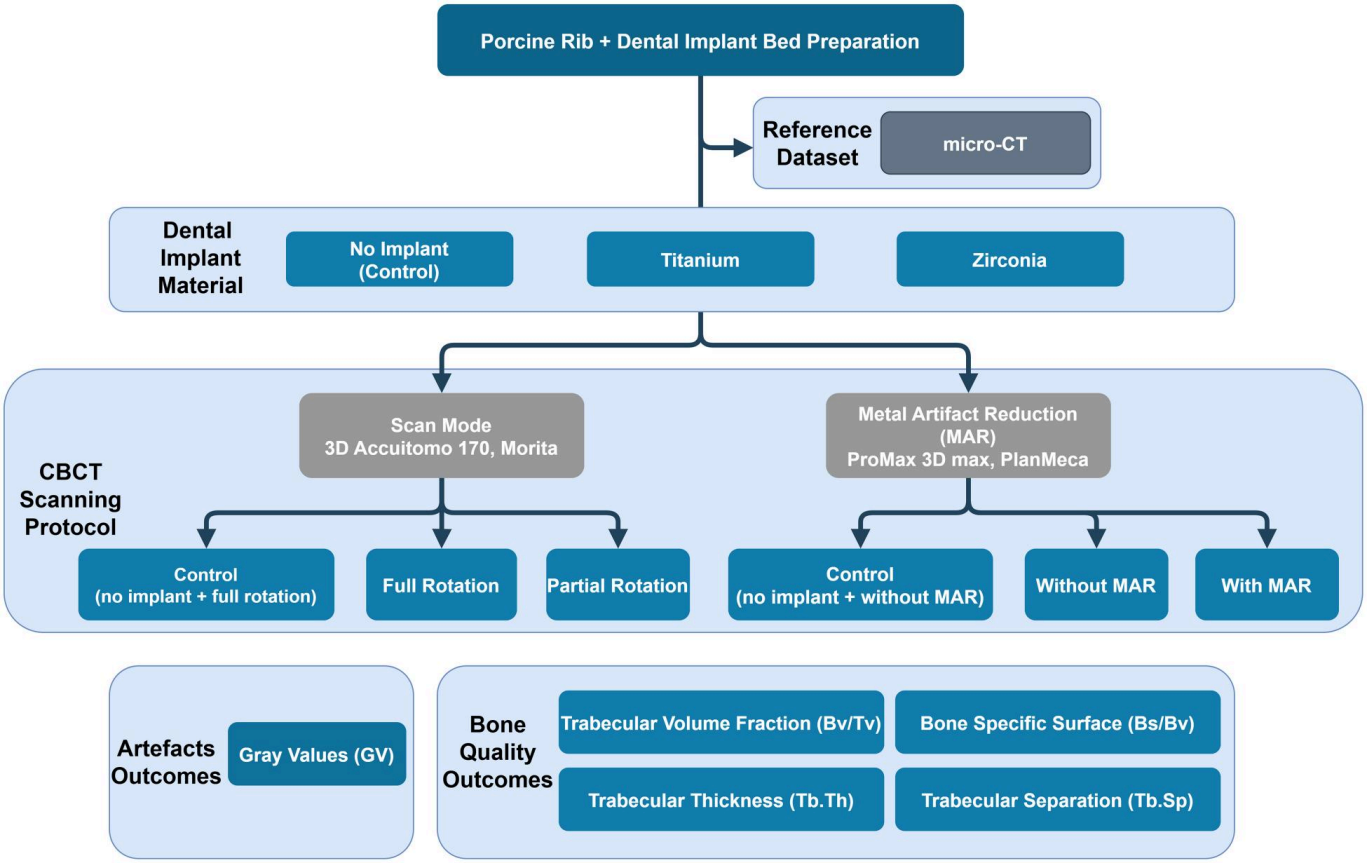
Study design flow chart.

Samples were clustered in groups of three specimens and positioned over a wax. Scans were performed adjusting the rotation mode (full rotation and half rotation) and the use of MAR algorithm (with MAR and without MAR), respectively (Figure 1).

### Measurements

Artefacts were assessed by measuring the SD of GV on peri-implant bone. In addition, bone qualitative measurements of dental implants were taken. All procedures, including data registration, bone segmentation, deviation of GV, and bone qualitative measurements were performed by two independent calibrated examiners (LB and HP) using Imalytics Preclinical software (Gremse-IT GmbH, Aachen, Germany).

**Figure 2.**
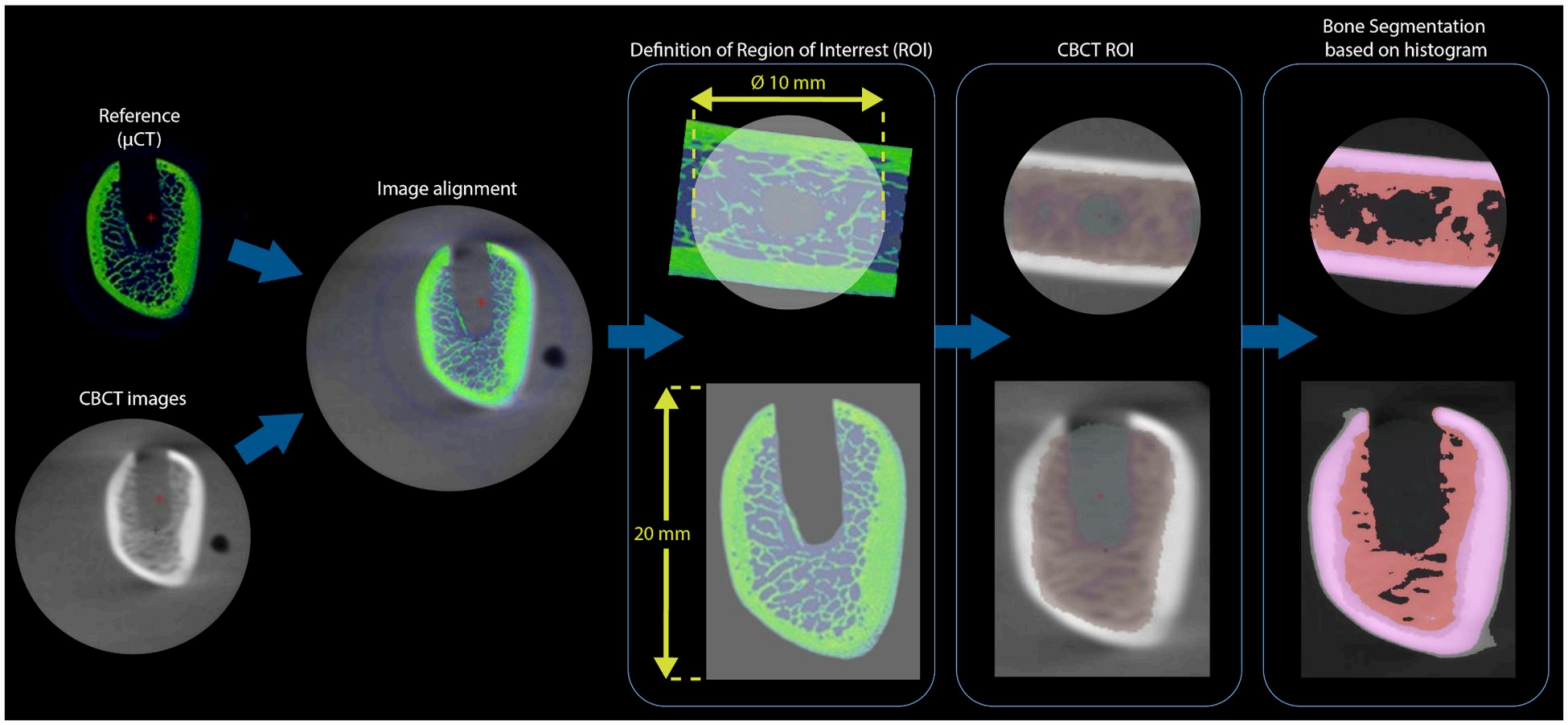
After the alignment of CBCT image to the reference dataset (µCT image), the region of interest was transferred to the CBCT image and segmented.

A cylindrical region of interest (ROI) measuring 10 mm of diameter and 20 mm of depth was created around dental implants. To avoid the influence of image quality on bone segmentation, all these steps were performed with reference dataset (µCT image). Each CBCT image was registered to the reference dataset by means of surface- and voxel-based fusion tools provided by the software (interactive alignment and marker-based fusion). At least three markers were placed in anatomical references on both images to guide the superimposition. After, matching was refined based on mean squared voxel differences. The ROI was then transferred from the reference dataset to the CBCT image, on which trabecular bone was segmented (Figure 2). GV deviation values were measured automatically by the software inside ROI.

**Figure 3.**
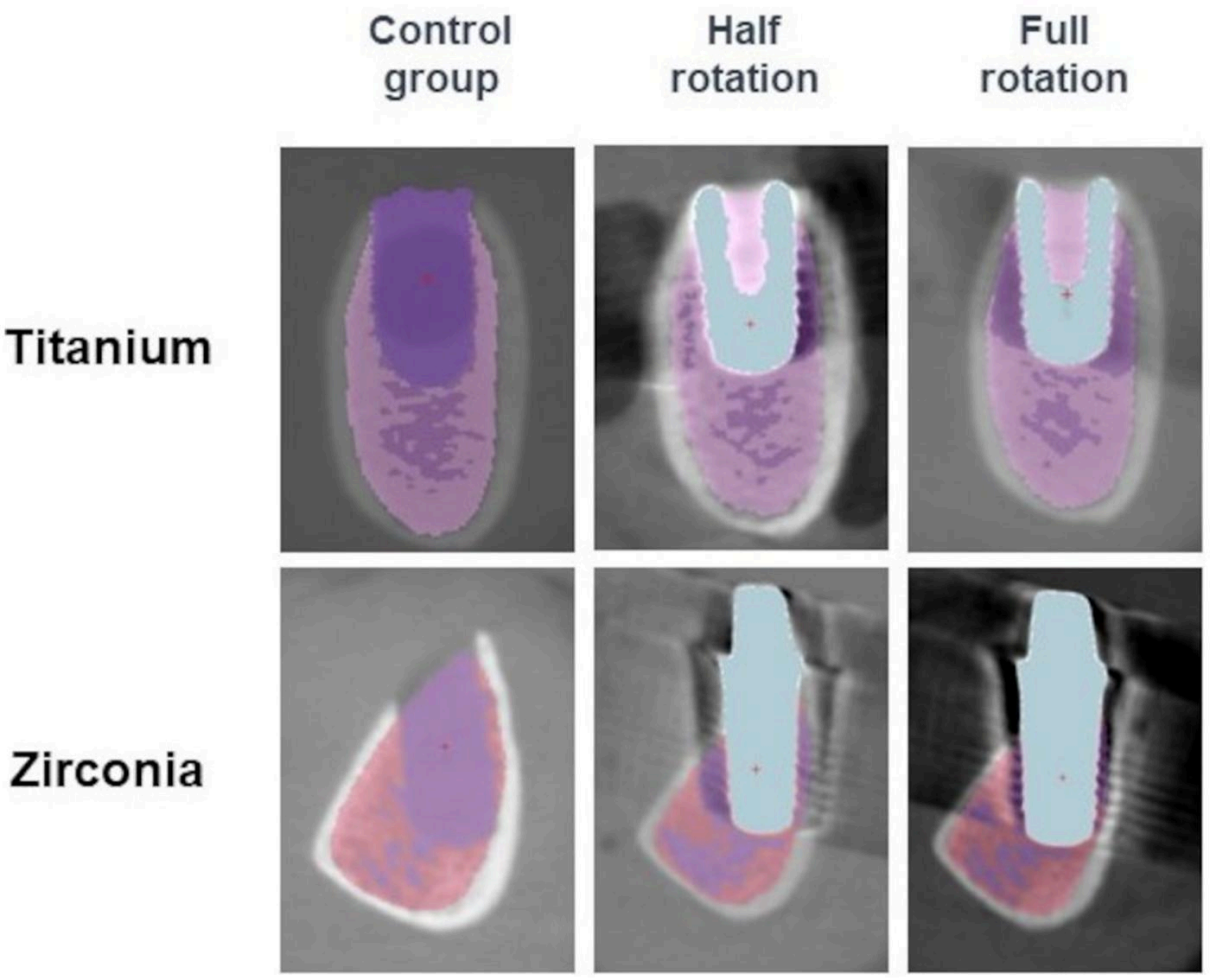
Trabeculae segmentation for the scan mode group.

**Figure 4.**
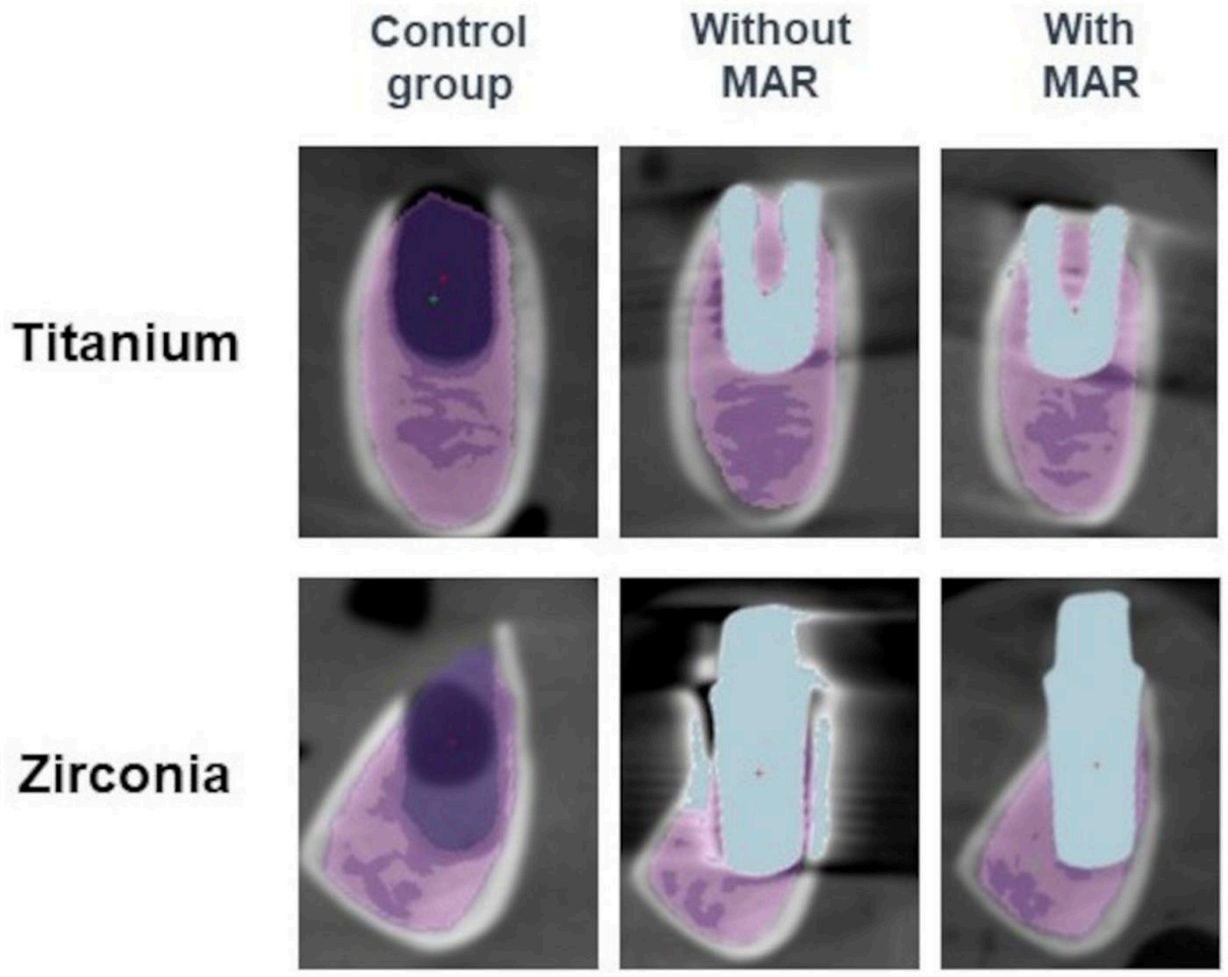
Trabeculae segmentation for the metal artefact reduction (MAR) group.

For bone qualitative measurements, trabeculae were additionally segmented based on threshold values (Figures 3 and 4). For this purpose, *a priori* information was calculated from the control groups specimen and used as reference for segmentation of the same bone samples containing dental implants. Bone quality was assessed by measuring the following parameters: trabecular volume fraction (Bv/Tv), bone specific surface (Bs/Bv), trabecular thickness (Tb.Th), trabecular separation (Tb.Sp).^22^

### Statistical analysis

Statistical analysis was performed with the software SPSS 28.0 (IBM, Armonk, NY, USA). Intra-class correlation (ICC) was used to calculate inter-agreement between examiners measurements and classified as excellent (ICC = 0.95). From these measurements, a mean value was calculated and defined as dependent variable. A within-ANOVA with Bonferroni correction was conducted. Dental implant material was considered a covariable, and a simple hierarchical model was applied having the control group as reference. Bone quality measurements were assessed by Spearman correlation test. All analyses were performed with a statistical significance level at *α* = .05.

## Results

### Artefacts assessment

#### Rotation mode

**Figure 5.**
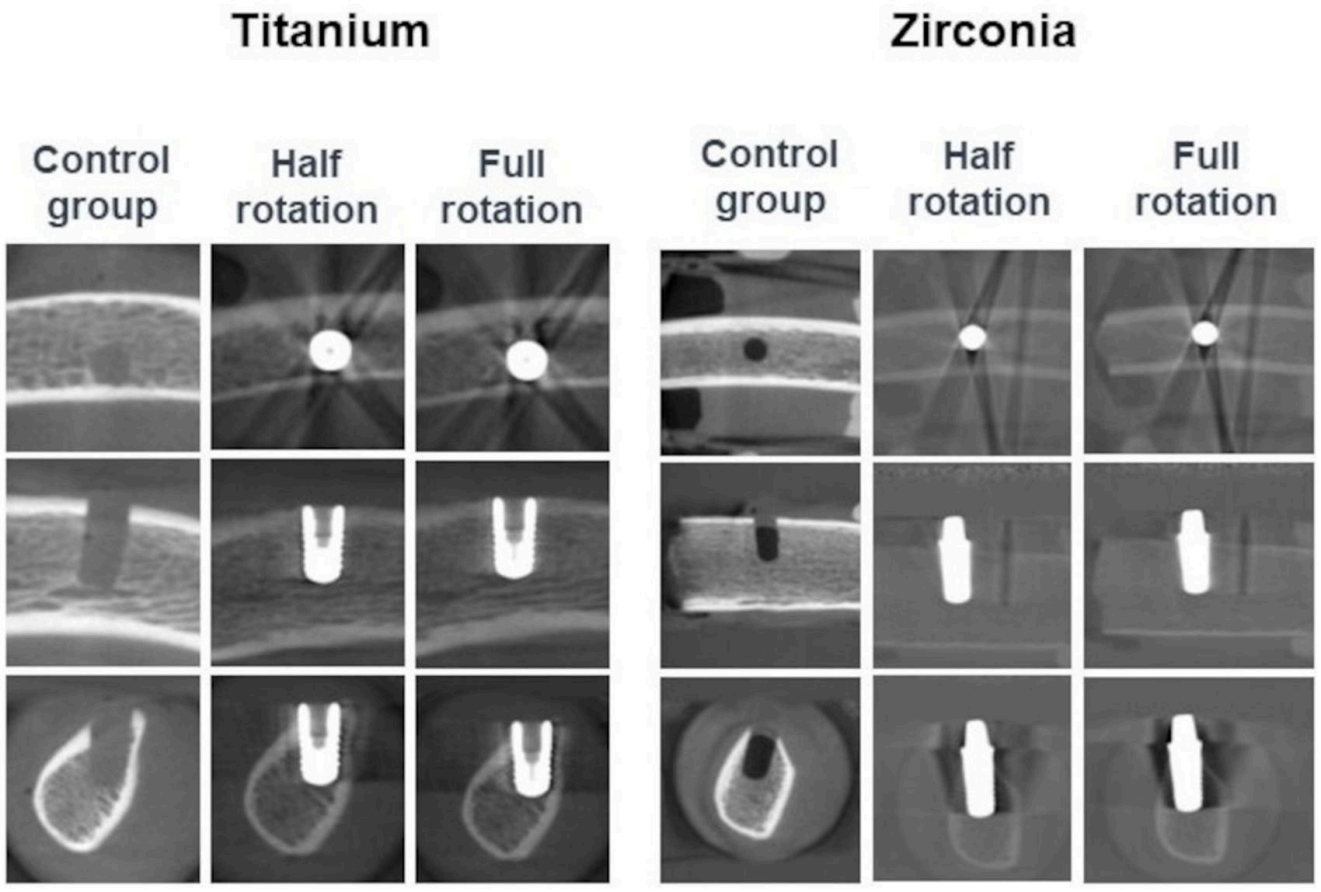
Illustrative images showing artefacts caused by titanium and zirconia dental implants in CBCT images acquired with different scan modes.

With regard to the rotation mode, no statistical difference was found between half and full-rotation modes (*P* = .82) for the same implant material (Figure 5). In comparison to the control group, GV deviation was higher in the presence of dental implants (*P* < .01), with the greatest artefacts displayed by zirconia implants (*P* < .01) (Appendices S2 and S3).

#### MAR algorithm

**Figure 6.**
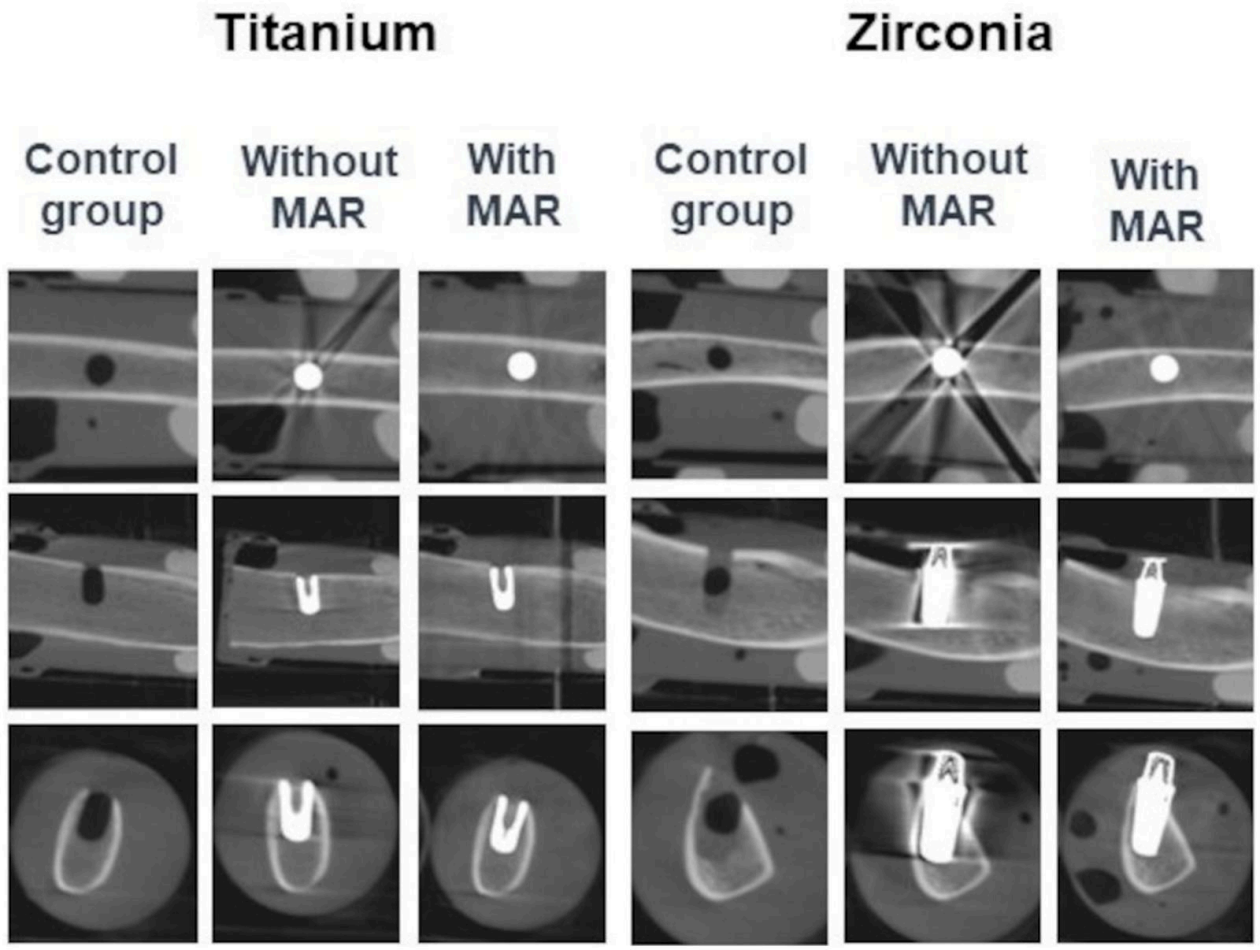
Artefacts caused by titanium and zirconia dental implants in CBCT images acquired according to the use of metal artefact reduction (MAR).

MAR algorithm reduced GV deviation of titanium and zirconia implants (Figure 6) to a mean value statistically similar to the control group (*P* = .20 and *P* = .31, respectively) (Appendices S2 and S3). Conversely, for both titanium and zirconia implants, significant GV deviations occurred when no MAR was applied, being statistically different from the control group (titanium: *P* = .013; zirconia: *P* < .01).

### Bone quality assessment

#### Rotation mode

**Figure 7.**
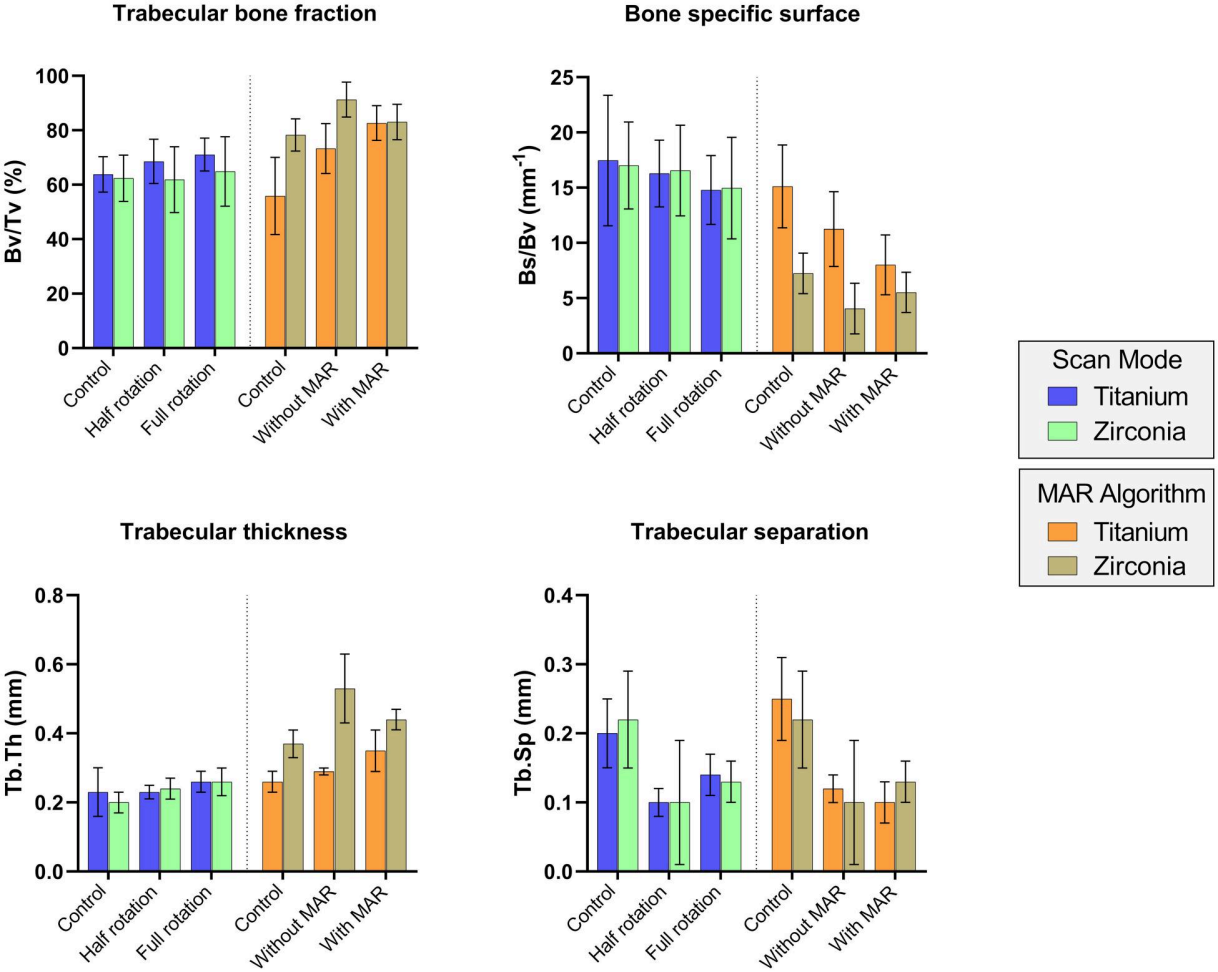
Quantitative assessment of bone quality for “scan mode” and “MAR algorithm”.

No statistically significant difference was found for bone measurements acquired with different rotation modes (Figure 7, Appendix S4). For both scan modes, Bv/Tv and Tb.Sp values of specimens containing dental implants showed no correlation with the control group (half rotation: *r* = 0.35 and 0.13; full rotation: *r* = 0.56 and 0.13, respectively). For Bs/Bv (half rotation: *r* = 0.53; full rotation: *r* = 0.73) and Tb.Th (half rotation: *r* = 0.42; full rotation: *r* = 0.63), only the full rotation mode was positively correlated with the control group. For all bone parameters, a high positive correlation was found between half and full rotation modes (Bv/Tv: *r* = 0.90; Bs/Bv: *r* = 0.92; Tb.Th: *r* = 0.93; Tb.Sp: *r* = 0.87).

#### MAR algorithm

For MAR algorithm, there was a statistically significant difference among the test and control groups, which were affected by dental implant material (Figure 7, Appendix S4). However, no difference was found between the groups with and without MAR. All bone parameters assessed on scans without MAR showed a positive correlation with the control group (Bv/Tv: *r* = 0.88; Bs/Bv: *r* = 0.90; Tb.Th: *r* = 0.84; Tb.Sp: *r* = 0.73). Conversely, scans with MAR showed a positive correlation to the control group only for Bs/Bv (*r* = 0.72) and Tb.Th (*r* = 0.72) parameters.

## Discussion

Bone quality assessment has been proposed for dental implant monitoring. However, in the presence of dental implants, image quality is compromised by artefacts, that make it difficult to evaluate peri-implant tissue.^23^ Our findings showed that bone measurements can be affected by the presence of artefacts caused by dental implants.

Artefacts occur due to different reasons. Scattering, for instance, is caused when X-rays deviate from their original path but still reach the detector, increasing the primary intensity and resulting in a faulty projection data. Conversely, beam-hardening artefacts are caused by high atomical number materials, as dental implants, which tend to absorb the lower energetic wavelengths emitted by X-rays, resulting in an increase of energy recording around the material. For both factors, artefacts show a streak-like appearance represented by dark and white areas along the projection lines.^24^

It is expected that a full rotation mode will provide a better image quality, since the quantity of basis images acquired during the scan is approximately 50% higher in comparison to the half-rotation mode. The basis images are used to provide information to the algorithm for image reconstruction. A higher number of basis images increases the contrast-noise ratio, however the appearance of artefacts is also more proeminent.^9^^,^^25^^,^^26^ Nonetheless, the findings of this study suggested that neither image quality nor bone quality assessment were affected by rotation mode. Considering that the radiation exposure is proportional to the number of acquired images, a reduced rotation mode is recommended to the assessment of peri-implant structures.^27^

Conversely, the present findings showed that the use of MAR might be beneficial to reduce artefacts, especially the one caused by zirconia implants. Nonetheless, it does not seem to enhance bone assessment. Scans without MAR showed a better performance for bone quality assessment, whereas MAR improved trabecular surface and thickness values. These results are in accordance with Vitulli et al,^15^ who showed that MAR affected the determination of bone parameters, which was directly influenced by the dental implant material. According to the authors, the increased susceptibility artefacts caused by zirconia implants could influence the determination of trabecular bone.

Different MAR approaches can be used to reduce artefacts,^16–33^ which can be applied prior or after image acquisiton.^21^^,^^28^ Although MAR have been evaluated by several studies,^6^^,^^13^^,^^29–32^ little is known about the image reconstruction method, and it is still unclear how it affects the diagnostic accuracy.^30^^,^^31^ It is suggested that a uniform threshold GV can be applied to surround metals and correct voxels with higher densities, resulting in a blooming appearance (spread-out appearance due to overestimation of the object’s density).^32^ This blurring effect does not represent real GV, and it might affect peri-implant bone assessment, which could justify discrepancies of this study when MAR algorithm was applied.

Limitations of this study should be addressed. The uniformity of GV is affected by both exposure parameters and appearance of artefacts.^8^^,^^33^ When determining a fixed threshold value for bone assessment, variations on trabeculae segmentation may occur and affect the measurements. In addition, peri-implant bone will be influenced by artefacts in different intensities, whether adjacent or apical to dental implants. However, the influence of the evaluated site over the GV and the location of metal artefacts was not considered in this analysis. Moreover, since Hounsfield (HU) units are not applicable in CBCT, these findings cannot be extended to different CBCT devices.^34^

Regarding bone samples, porcine ribs were chosen due to its similarity to human maxilla bone.^35^ However, the influence of different types of bone quality (I-IV) over the appearance of artefacts was not considered on the analysis. Thus, these results could differ according to the jaw and region evaluated.

In addition, soft tissue was removed from the specimens to minimize interferences in MRI of bone tissues, however differing from clinical settings with patients.^36^ Likewise, the partial object effect caused by surrounding soft and hard tissues was not considered in this study.

Future studies should focus on the assessment of image quality by using the aforementioned CBCT protocols with an acceptable radiation dose to the peri-implant bone assessment. An alternative could be the reduction of the field of view, in order to create images with high resolution and lower noise.

## Conclusion

- Bone quality assessment was affected by the presence of artefacts caused by dental implants.
- Rotation mode did not affect the appearance of artefacts and bone qualitative measurements.
- MAR was able to minimize artefacts, however, it did not improve the accuracy of bone quality measurements.

## Supplementary Material

twaf003_Supplementary_Data
