# Supplementary material for: Bone quality assessment around dental implants in cone-beam CT images: effect of rotation mode and metal artefact reduction tool
Source: Dentomaxillofac Radiol. 2025 Feb 13;54(4):286–93. doi: 10.1093/dmfr/twaf003 (PMC12038231; doi:10.1093/dmfr/twaf003)
Supplement: twaf003_Supplementary_Data [file twaf003_supplementary_data.zip › twaf003_Supplementary_Data/Appendix 1_v3.docx]

Appendix 1. Dental implants characteristics.

| **Sample** | **Composition** | **Dimension** | **Geometry** | **Material; Brand** |
| --- | --- | --- | --- | --- |
| Titanium Grade 4 | Ti, O ≤ 0.4%, Fe 0.25–0.5%, N ≤ 0.05%, C ≤ 0.10%, H ≤ 0.012%, | 4.1 x 8 mm | Two-piece bone level | Bone Level, Institute Straumann AG, Basel, Switzerland |
| Yttria-stabilized tetragonal zirconia (Y-TZP) | ZrO2 + HfO2 + Y2O3≥99.0%, Y2O3>4.5% to ≤6.0%, HfO2≤5.0%, residuals≤1.0%, | 4.1 x 8 mm | One-piece tissue level | Pure Ceramic, Institute Straumann AG, Basel, Switzerland |
