# Supplementary material for: Bone quality assessment around dental implants in cone-beam CT images: effect of rotation mode and metal artefact reduction tool
Source: Dentomaxillofac Radiol. 2025 Feb 13;54(4):286–93. doi: 10.1093/dmfr/twaf003 (PMC12038231; doi:10.1093/dmfr/twaf003)
Supplement: twaf003_Supplementary_Data [file twaf003_supplementary_data.zip › twaf003_Supplementary_Data/Appendix 2_v4.docx]

Appendix 2). Gray values ± standard deviation (SD) for “scan mode” and “MAR algorithm”.

| **Scan mode** | | |
| --- | --- | --- |
| **Group** | Dental implant | Gray values ± SD |
| **Control group** | Ti | 261.19 ± 18.66 |
|  | Zi | 241.77 ± 20.95 |
|  | Total | 251.48 ± 21.46** |
| **Half rotation** | Ti | 418.06 ± 90.36 |
|  | Zi | 723.20 ± 122.78 |
|  | Total | 570.63 ± 189.62 |
| **Full rotation** | Ti | 385.02 ± 63.43 |
|  | Zi | 736.79 ± 148.32 |
|  | Total | 560.91 ± 213.49 |
| **MAR algorithm** | | |
| **Group** | Dental implant | Gray values ± SD |
| **Control group** | Ti | 458.99 ± 35.81 |
|  | Zi | 483.41 ± 78.88 |
|  | Total | 471.207 ± 59.78 |
| **Without MAR** | Ti | 513.40 ± 16.70 |
|  | Zi | 1067.74 ± 285.18 |
|  | Total | 790.57 ± 347.71 ** |
| **With MAR** | Ti | 484.51 ± 42.37 |
|  | Zi | 593.80 ± 124.40 |
|  | Total | 539.16 ± 105.39 |

Ti: titanium, Zi: zirconia, ** means statistical significant difference at α=0.05.
