# Supplementary material for: Bone quality assessment around dental implants in cone-beam CT images: effect of rotation mode and metal artefact reduction tool
Source: Dentomaxillofac Radiol. 2025 Feb 13;54(4):286–93. doi: 10.1093/dmfr/twaf003 (PMC12038231; doi:10.1093/dmfr/twaf003)
Supplement: twaf003_Supplementary_Data [file twaf003_supplementary_data.zip › twaf003_Supplementary_Data/Appendix 3_v4.docx]

Appendix 3). Within ANOVA for “scan mode” and “MAR algorithm”:

|  | **Sum of squares** | **df** | **Mean square** | **F-value** | **p-value** |
| --- | --- | --- | --- | --- | --- |
| **Scan mode** | 790797.472 | 2 | 395398.73 | 53.353 | < 0.001 |
| **MAR algorithm** | 679271.552 | 2 | 339635.77 | 17.760 | <0.001 |

df= degree of freedom.
