# Supplementary material for: Bone quality assessment around dental implants in cone-beam CT images: effect of rotation mode and metal artefact reduction tool
Source: Dentomaxillofac Radiol. 2025 Feb 13;54(4):286–93. doi: 10.1093/dmfr/twaf003 (PMC12038231; doi:10.1093/dmfr/twaf003)
Supplement: twaf003_Supplementary_Data [file twaf003_supplementary_data.zip › twaf003_Supplementary_Data/Appendix 4_v4.docx]

Appendix 4). Qualitative assessment of bone for “scan mode” and “MAR algorithm”.

|  | **Scan mode** | **BvTv** | **BsBv** | **TbTh** | **TbSp** |
| --- | --- | --- | --- | --- | --- |
| **Titanium** | Control group | 63.80 ± 6.53 | 17.46 ± 5.90 | 0.23 ± 0.07 | 0.20 ± 0.05 |
|  | Half rotation | 68.57 ± 8.11 | 16.28 ± 3.02 | 0.23 ± 0.02 | 0.10 ± 0.02 |
|  | Full rotation | 71.08 ± 6.07 | 14.79 ± 3.12 | 0.26 ± 0.03 | 0.14 ± 0.03 |
| **Zirconia** | Control group | 62.34 ± 8.50 | 17.01 ± 3.94 | 0.20 ± 0.03 | 0.22 ± 0.07 |
|  | Half rotation | 61.86 ± 12.07 | 16.55 ± 4.11 | 0.24 ± 0.03 | 0.10 ± 0.09 |
|  | Full rotation | 64.85 ± 12.75 | 14.96 ± 4.60 | 0.26 ± 0.04 | 0.13 ± 0.03 |
|  | p-value | **0.43** | **0.81** | **0.16** | **0.90** |
|  | **MAR algorithm** | **BvTv** | **BsBv** | **TbTh** | **TbSp** |
| **Titanium** | Control group | 55.88 ± 14.16 | 15.11 ± 3.75 | 0.26 ± 0.03 | 0.25 ± 0.06 |
|  | Without MAR | 73.30 ± 9.14 | 11.25 ± 3.38 | 0.29 ± 0.01 | 0.12 ± 0.02 |
|  | With MAR | 82.66 ± 6.35 | 8.01 ± 2.70 | 0.35 ± 0.06 | 0.10 ± 0.03 |
| **Zirconia** | Control group | 78.29 ± 5.93 | 7.24 ± 1.83 | 0.37 ± 0.04 | 0.15 ± 0.02 |
|  | Without MAR | 91.28 ± 6.40 | 4.06 ± 2.29 | 0.53 ± 0.10 | 0.09 ± 0.02 |
|  | With MAR | 83.07 ± 6.50 | 5.52 ± 1.83 | 0.44 ± 0.03 | 0.13 ± 0.02 |
|  | p-value | **<0.01** | **<0.01** | **<0.01** | **<0.01** |

Bv/Tv = trabecular volume fraction; Bs/Bv = bone specific surface; Tb.Th = trabecular thickness; Tb.Sp = trabecular separation. MAR = Metal artifact reduction.
